# Can Chloroform Be Used to Facilitate Robbery?

**Published:** 1872-05

**Authors:** 


### Books Reviewed.

*Can Chloroform be used to facilitate Robbery?* By STEPHEN ROGERS, M. D. President of the New York Medico-Legal Society. Reprint from Journal of Psychological Medicine.

In this pamphlet, which constitutes a paper read before the New York Medico-Legal Society, Dr. Rogers gives an account of many of the cases of Robbery, etc., said to have been performed with the aid of chloroform. Many of the cases seem to be simply willful deception on the part of those robbed, others arise from ignorance of the properties of chloroform; while not a few are chargeable simply to exaggerated rumor or report. Dr. Rogers' paper is an able criticism on the reported cases of Robbery with the aid of chloroform. In conclusion he challenges the production of proof in one single instance of the successful use of chloroform on the human subject to facilitate Robbery.

